# Continuous degradation of maltose by enzyme entrapment technology using calcium alginate beads as a matrix

**DOI:** 10.1016/j.bbrep.2015.09.025

**Published:** 2015-10-08

**Authors:** Muhammad Asif Nawaz, Haneef Ur Rehman, Zainab Bibi, Afsheen Aman, Shah Ali Ul Qader

**Affiliations:** The Karachi Institute of Biotechnology and Genetic Engineering (KIBGE), University of Karachi, Karachi 75270, Pakistan

**Keywords:** Maltase, Biodegradation, Immobilization, Calcium alginate beads, Thermal stability, Enzyme activity

## Abstract

Maltase from *Bacillus licheniformis* KIBGE-IB4 was immobilized within calcium alginate beads using entrapment technique. Immobilized maltase showed maximum immobilization yield with 4% sodium alginate and 0.2 M calcium chloride within 90.0 min of curing time. Entrapment increases the enzyme–substrate reaction time and temperature from 5.0 to 10.0 min and 45 °C to 50 °C, respectively as compared to its free counterpart. However, pH optima remained same for maltose hydrolysis. Diffusional limitation of substrate (maltose) caused a declined in *V*_*max*_ of immobilized enzyme from 8411.0 to 4919.0 U ml^−1^ min^−1^ whereas, *K*_*m*_ apparently increased from 1.71 to 3.17 mM ml^−1^. Immobilization also increased the stability of free maltase against a broad temperature range and enzyme retained 45% and 32% activity at 55 °C and 60 °C, respectively after 90.0 min. Immobilized enzyme also exhibited recycling efficiency more than six cycles and retained 17% of its initial activity even after 6th cycles. Immobilized enzyme showed relatively better storage stability at 4 °C and 30 °C after 60.0 days as compared to free enzyme.

## Highlights

• Immobilized maltase showed maximum immobilization yield with 4% sodium alginate and 0.2 M calcium chloride.
• Immobilized maltase showed maximum relative activity with 2.0 mm beads size.
• Immobilization also increased the stability of free maltase against a broad temperature range.
• Immobilized enzyme also exhibited admirable recycling efficiency up to 06 reaction cycles that retained 17% of its initial activity even after six cycles.

## Graphical abstract

**Figure:**
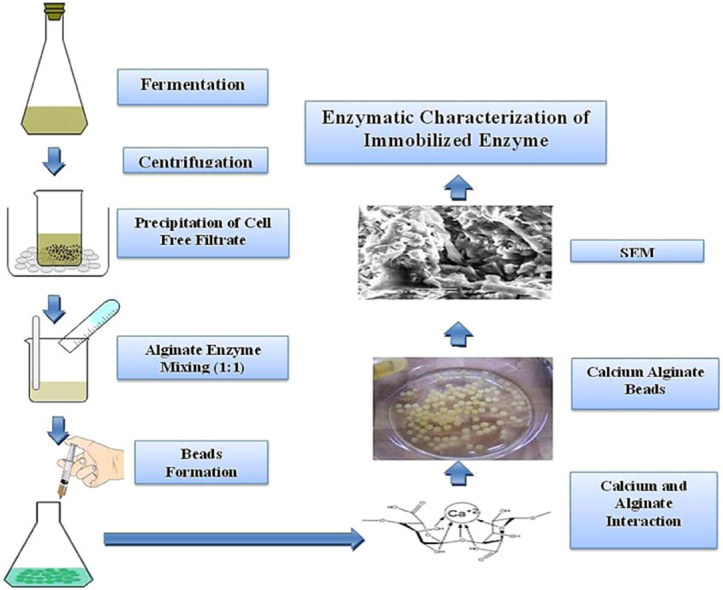

## 1 Introduction

Maltase [E.C 3.2.1.20] is an enzyme that catalyzes the hydrolytic process of α-1→4 glycosidic linkages and yields α-D-glucose as an end product. Maltase is used in different industries like food, brewing, distilling and pharmaceutical industry [1]. Catalytic property of an enzyme is one of the critical aspects for its commercialization and in some cases free enzymes are unstable to fulfill the requirement of industries due to low operational stability and recovery [2]. Various techniques such as protein engineering, chemical modification and immobilization have been investigated to overcome the limitation of enzymes for industrial bioreactors [3], [4], [5]. Immobilization is an encouraging approach that not only enhances the stability of enzymes but also ensures the reusability of enzymes for various industrial bioprocesses [6], [7], [8], [9]. Different immobilization techniques including covalent binding, adsorption, crosslinking and entrapment are used for continuous use of different enzymes on industrial scale. Among them, entrapment confines the enzyme within the structured matrix space and create negligible impact on its catalytic properties [10], [11]. There are various matrices such as alginate, polyacrylamide and agar–agar are employed for enzyme immobilization. Alginate is an anionic polysaccharide distributed widely in the cell walls of brown algae and commonly used for the formation of beads capable of entrapping different macromolecules in the form of calcium-alginate beads [12], [13], [14]. Calcium alginate is a cost effective and biocompatible matrix for the entrapment of different enzymes including α-amylase, protease and pectinase [15], [16].

In the current study, maltase from *B. licheniformis* KIBGE-IB4 was immobilized using entrapment technique within calcium alginate beads.

## 2 Materials and methods

### 2.1 Induction of bacterial isolate for maltase production

Wheat starch was used for the induction of previously isolated *B. licheniformis* KIBGE-IB4 for extracellular maltase production. Culture was incubated in optimized fermentation medium: wheat starch (2.5%), peptone (1.0%), yeast extract (0.2%), meat extract (0.4%), K_2_HPO_4_ (0.3%) and KH_2_PO_4_ (0.1%) at 37 °C and pH-7.0 for 48 h [17]. The cells were harvested by centrifugation at 40,248×*g* for 15.0 min at 4 °C. Cell free filtrate (CFF) was precipitated using 40% saturation of ammonium sulfate on ice bath and kept for 24 h at 4 °C. The obtained precipitates were dissolved in potassium phosphate buffer (100.0 mM, pH-6.5) and dialyzed against same buffer for 24 h at 4 °C and used for immobilization within calcium alginate beads.

### 2.2 Maltase immobilized calcium alginate beads formation

Partially purified maltase was mixed with equal volume (1:1) of sodium alginate (4.0%) and added drop wise into calcium chloride solution (0.2 M) with constant shaking on ice bath. Immobilized enzyme and control beads were hardened by storing them into fresh 0.2 M calcium chloride solution for 90.0 min at 4 °C. The beads were washed with deionized water and potassium phosphate buffer to remove any unbound enzyme molecules.

### 2.3 Enzyme assay for free and immobilized maltase

The enzyme activity of both free and immobilized maltase was determined by GOD–PAP method [18], [19] using glucose and maltose as a standard and substrate respectively.

One unit of maltase was defined as the “*amount of enzyme required to release 1.0 µmol of glucose per minute under the standard assay conditions* ”.

### 2.4 Effect of sodium alginate and calcium chloride concentration on beads formation

Different concentrations of sodium alginate (1.0–6.0%) and calcium chloride (0.1–0.5 M) were used to attain the maximum immobilization yield of maltase with stable beads structure.

### 2.5 Effect of curing time on percent immobilization yield of maltase

The influence of curing time on percent immobilization of enzyme was examined by keeping beads in 0.2 M calcium chloride solution for different time intervals ranging from 30.0 to 180.0 min. Beads were washed with double deionized water and potassium phosphate buffer (pH-6.5).

### 2.6 Effect of bead size on the activity of immobilized maltase

The effect of beads size on the catalytic activity of immobilized maltase was determined by preparing beads having different diameters ranging from 1 to 5 mm.

### 2.7 Effect of reaction time on the catalytic activity of free and immobilized enzyme

The effect of reaction time on the catalytic activity of free and immobilized maltase was studied by performing the enzyme assay at different time intervals keeping the reaction temperature, pH and substrate constant.

### 2.8 Effect of temperature on the catalytic activity of free and immobilized enzyme

The effect of temperature on the catalytic activity of free and immobilized maltase was studied by varying the enzyme–substrate reaction temperature ranging from 30 °C to 60 °C, keeping other parameters constant.

### 2.9 Effect of pH on the catalytic activity of free and immobilized enzyme

The influence of pH on the catalytic activity of free and immobilized maltase was investigated by performing the enzyme assay at different pH ranging from 5.0 to 8.0. Different buffers such as acetate buffer (pH: 5.0–6.0), citrate buffer (pH: 5.0–6.0) and potassium phosphate buffer (pH: 7.0–8.0) with same ionic strength (100.0 mM) were used.

### 2.10 Substrate kinetics of free and immobilized enzyme

The kinetic parameters such as *K*_*m*_ and *V*_*max*_ of free and immobilized maltase were determined using Lineweaver–Burk plot by performing the enzyme assay at different substrate concentrations (2.5–50 mM).

### 2.11 Thermal stability of free and immobilized enzyme

Thermal stability of free and immobilized maltase were examined by keeping them at different temperatures such as 40 °C, 45 °C, 50 °C, 55 °C and 60 °C for different time intervals (30.0–180.0 min). Sample aliquots were retrieved after every 30.0 min and enzyme assay was performed at 50 °C for 10 min.

### 2.12 Storage stability of free and immobilized enzyme

Storage stability of free and immobilized maltase was determined by storing them at different temperatures (4 °C and 30 °C) for 60 days. Control and immobilized enzyme beads were placed in 0.1 M potassium phosphate buffer (pH-6.5) and stored at aforementioned temperatures. Percent residual activity was calculated by considering activity of control as 100%.

### 2.13 Recycling efficiency of immobilized enzyme

Reusability of immobilized maltase was investigated by using 0.5 g of beads for several reaction cycles. Beads were washed with phosphate buffer (pH-7.0) after each reaction cycle and fresh substrate was mixed for next reaction. Percent residual activity was calculated by considering the activity of first run as 100%.

### 2.14 Scanning electron microscopy of beads with and without immobilized enzyme

Scanning electron microscopy was used to study the morphological changes of beads with and without immobilized maltase. For this purpose, beads were allowed to dry at 37 °C for 24 h and coated with gold using auto coater (Jeol Japan, Model JFC-1500). These gold coated samples were subjected to scanning electron microscope (SEM) (Jeol Japan, Model JSM 6380 A) for morphological analysis at different magnification power range (2500× and 5000×).

## 3 Results and discussion

**Fig. 1:**
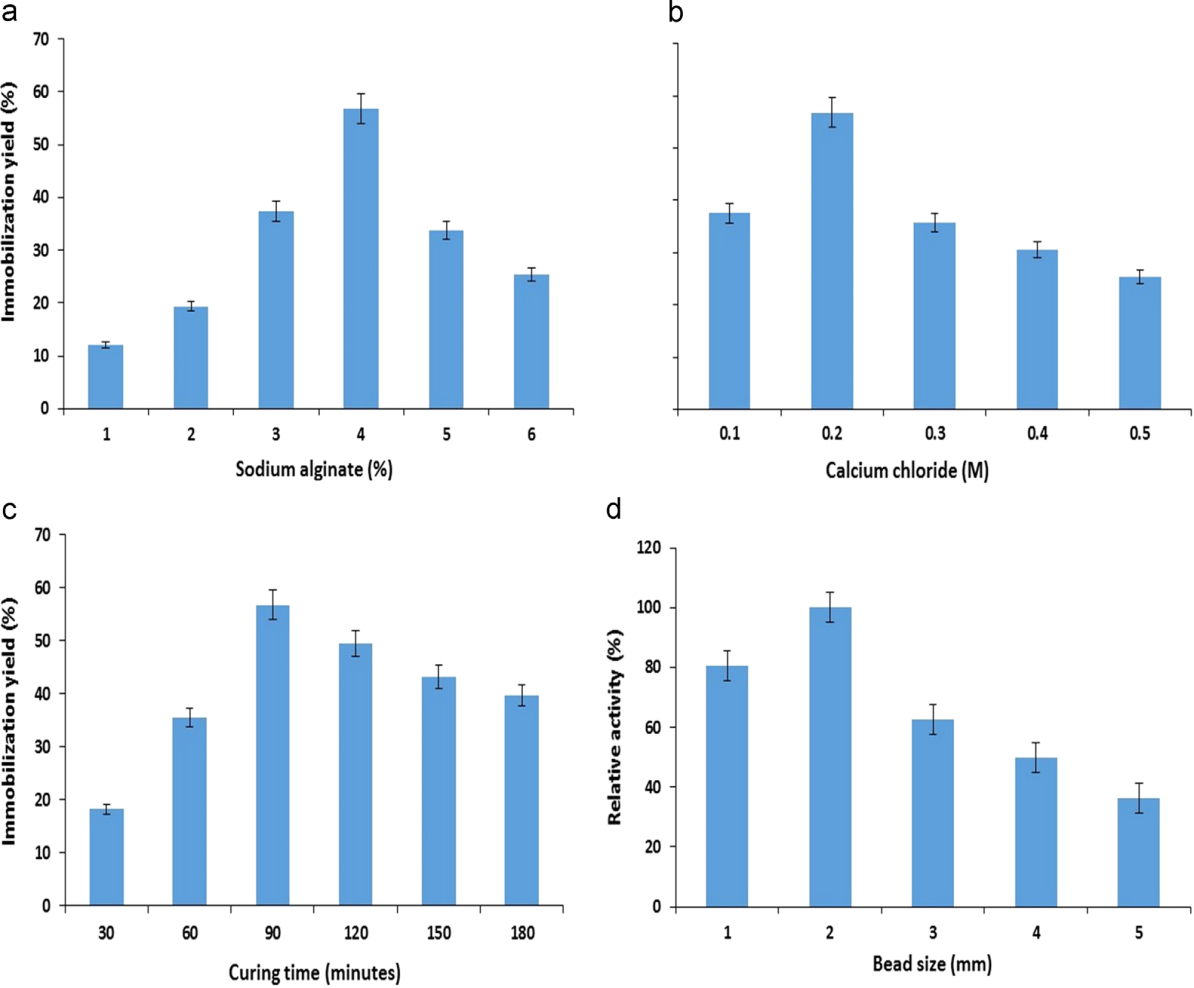
Effect of sodium alginate (A), calcium chloride (B), concentrations and (C) curing time (D) beads size on the immobilization of maltase from *B. licheniformis* KIBGE-IB4 (means±S.E., *n*=6).

Bacterial isolates are preferred to use at commercial level due to short fermentation time and selective product formation but free enzyme always faces problem due to difficult recovery from reaction mixture which also decreases the product purity. Immobilization is a supportive technique which not only permits easy recovery of product and enzyme from reaction mixture but also improves the thermal and operational stability of an enzyme. Effect of sodium alginate concentration on entrapment of maltase was studied different concentrations of sodium alginate. It was observed that the percent immobilization yield of maltase was found maximum at 4.0% sodium alginate (Fig. 1A) and as the concentration of sodium alginate increased from maximum level, immobilization efficiency decreased which might be due to the formation of beads having small pore size that causes hindrance to enter the substrate in calcium alginate beads and to react with immobilized enzyme. However, low concentration of sodium alginate resulted in the formation of soft and fragile beads with large pore size which resulted in the increased enzyme leakage during washing step [20], [21]. Therefore proper concentration of sodium alginate is necessary for maximum immobilization of enzyme.

It was observed that calcium chloride (0.2 M) provided more stabilized beads with maximum percent immobilization yield (Fig. 1B). The percent immobilization of maltase was decreased when concentration of calcium chloride increased or decreased beyond optimum level. High concentration of calcium chloride causes change in pH which might be responsible to decrease the catalytic activity of enzyme in microenvironment of beads. Immobilized maltase showed relatively high immobilization yield as compared to immobilized pectinase, while lower than immobilized α-amylase within same matrix [3], [15].

The curing time of calcium alginate beads was also investigated for synthesis of stable beads structure. It was found that the percent immobilization yield increased with time and maximum immobilization yield was achieved after 90 min (Fig. 1C). It was previously reported that the probability of cross linkages formation increased between the matrix and enzyme when beads exposed with calcium chloride solution for relatively long time period [22]. However, it was also found that immobilization yield reduced due to prolonged curing time of beads which might be due to the enzyme leakage. Fragile beads are formed when beads are not exposed to calcium chloride solution for optimum time [14]. An appropriate bead size is also significant factor that affects the catalytic efficiency of immobilized enzyme and determines the suitability of reactor configuration [23]. It was observed that beads having 2.0 mm diameter size showed maximum catalytic activity as compared to other beads sizes (Fig. 1D). Catalytic activity was declined as diameter size of beads increased or decreased beyond optimum size. It was assumed that the beads size of 2.0 mm might provide more favorable conditions in terms of substrate and product diffusion from the matrix as compared to other sizes.

**Fig. 2:**
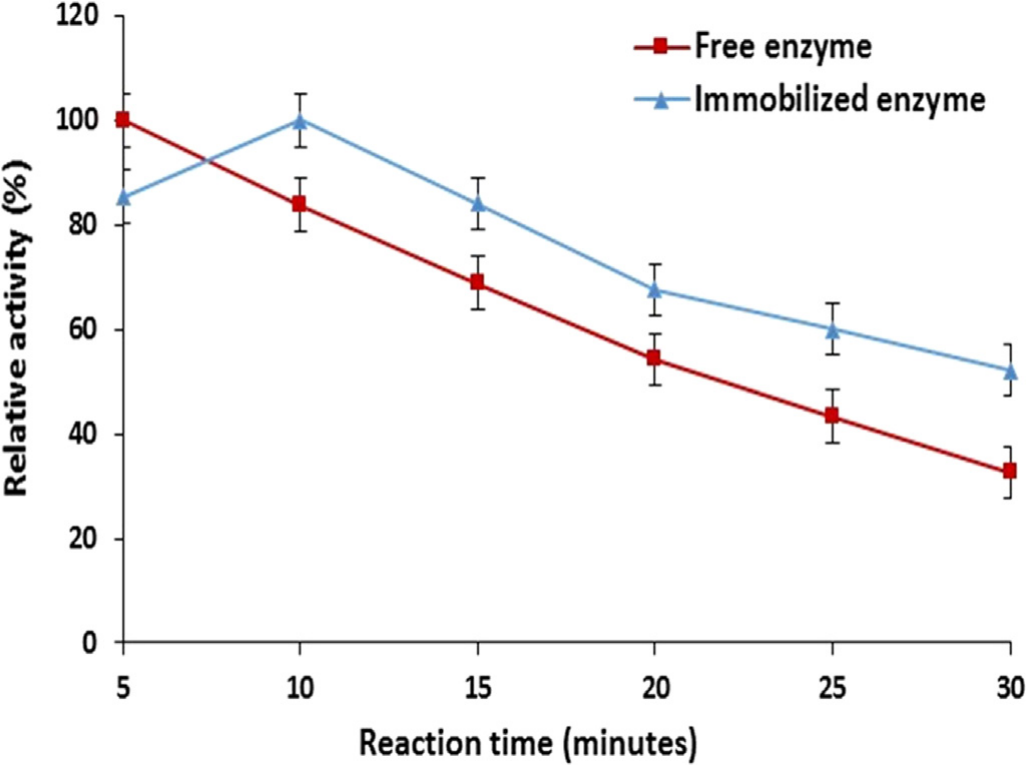
Effect of reaction time on the catalytic activity of calcium alginate immobilized maltase (means±S.E., *n*=6).

The impact of enzyme–substrate reaction time was also investigated on the catalytic activity of free and immobilized maltase. It was found that reaction time of immobilized maltase increased and maximum activity was achieved after 10.0 min as compared to free enzyme having maximum activity at 5.0 min at 50 °C (Fig. 2). This shift in reaction time might be due to the diffusion limitation of substrate into microenvironment of immobilized beads in order to reach the active site of enzyme. Similar findings have been reported in case of dextransucrase when immobilized within same support [24].

**Fig. 3:**
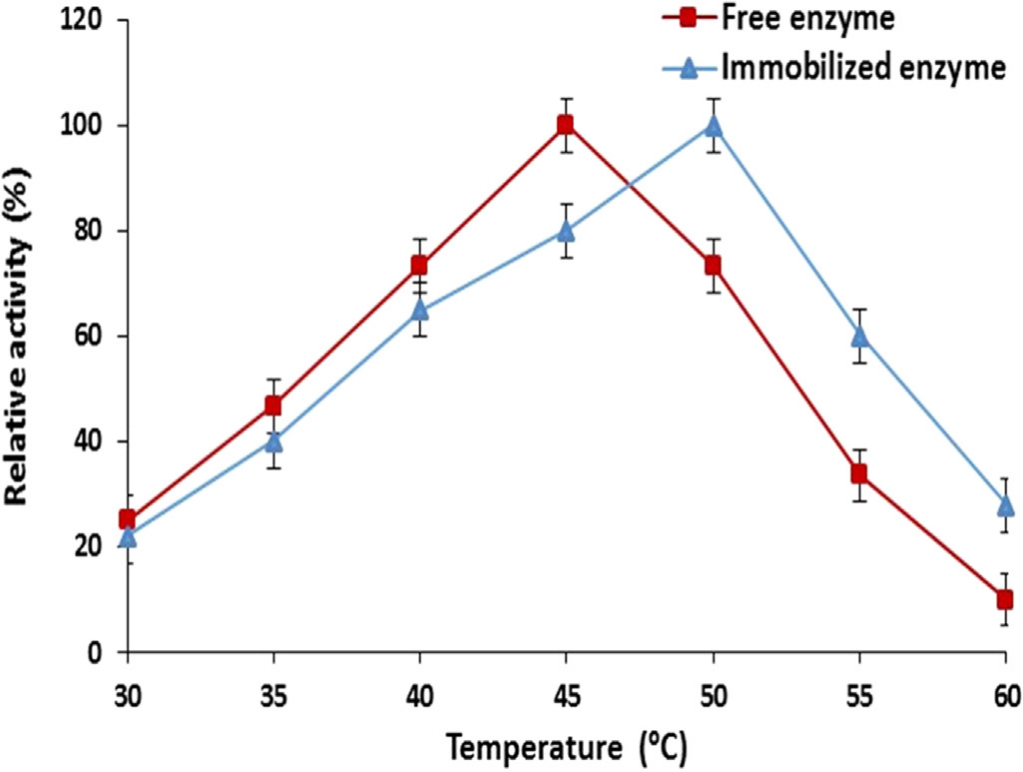
Effect of incubation temperature on the catalytic activity of calcium alginate immobilized maltase (means±S.E., *n*=6).

Effect of reaction temperature on the catalytic activity of free and immobilized maltase was carried out at 30–60 °C for 10 min and it was revealed that the immobilized maltase showed maximum relative activity at 50 °C as compared to free maltase having optimum activity at 45 °C (Fig. 3). This shift in temperature optima might occur due to the impairment of hydrophobic and secondary interactions of the enzyme molecules within the matrix which improved at higher temperature to attain the maximum catalytic activity. It has been reported earlier that the immobilized endo-β-1,4-xylanase showed maximum relative activity at 60 °C which was 10 °C increase in temperature as compared to free enzyme [25]. Same finding was reported when endo (1→4) β-D-glucanase immobilized within agar–agar matrix using same technique where temperature optima was shifted from 60 °C to 70 °C after immobilization [26].

**Fig. 4:**
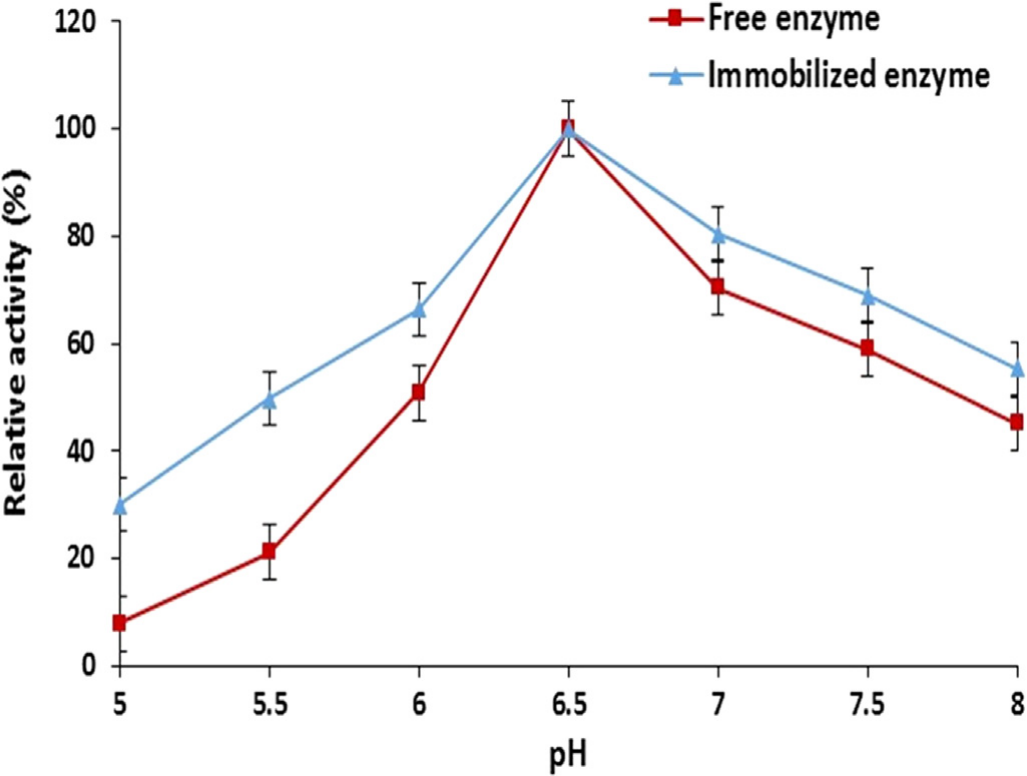
Effect of reaction pH on the catalytic activity of calcium alginate immobilized maltase (means±S.E., *n*=6).

Effect of pH on the catalytic activity of free and immobilized maltase was performed at different pH levels (5.0–8.0) and it was observed that entrapment of maltase did not change the optimum pH of maltase activity and maximum relative activity was obtained at pH-6.5 (Fig. 4). It was noted that immobilized maltase extended its activity at broad pH range as compared to free enzyme.

**Fig. 5:**
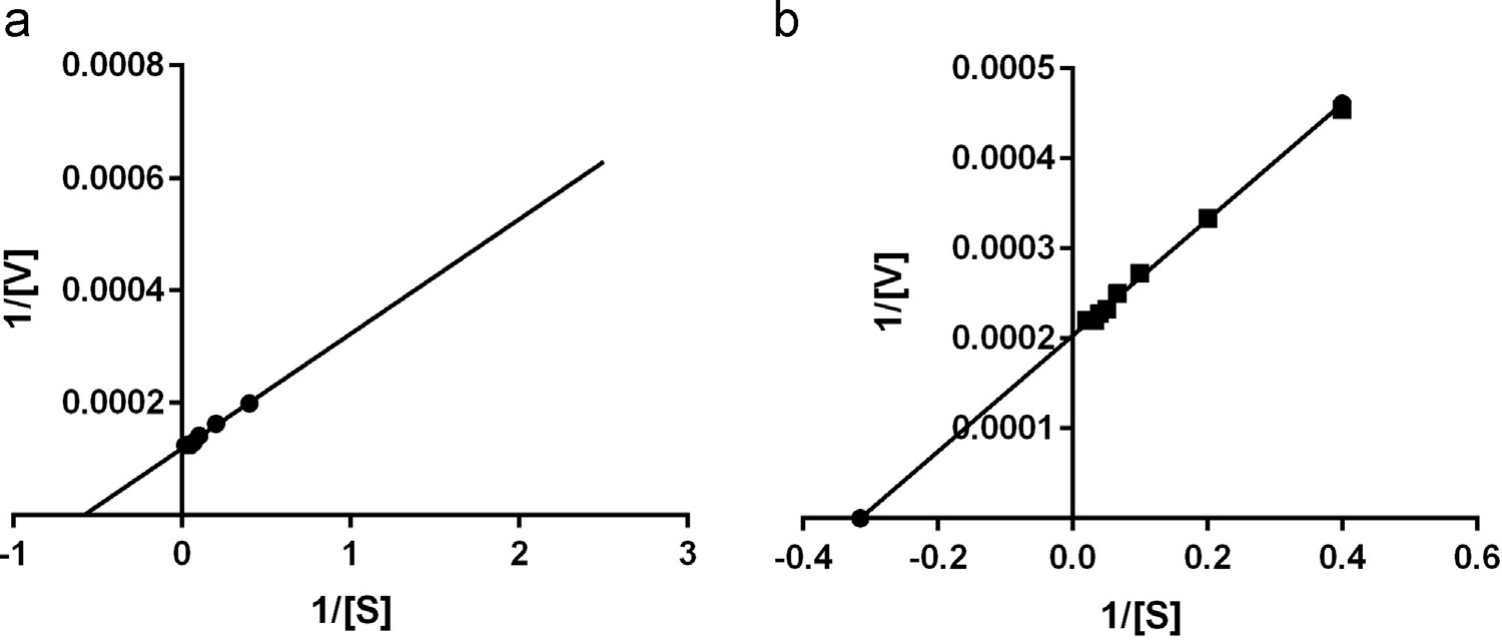
Lineweaver–Burk plots of (a) soluble and (b) immobilized maltase.

*K*_*m*_ and *V_max_* values of both free and immobilized maltase were calculated using Lineweaver–Burk plot by performing the enzymatic assay using different substrate concentrations. It was found that the *K_m_* value of maltase was apparently increased from 1.71 to 3.17 mM ml^−1^ and *V_max_* decreased from 8411 U ml^−1^ min^−1^ to 4919 U ml^−1^ min^−1^ after immobilization (Fig. 5). Increase in *K_m_* and decrease in *V_max_* values might be due to the hindrance of substrate to penetrate in matrix for enzyme–substrate reaction. Similar finding has been reported when maltase was immobilized within agar–agar support using same technique [27].

**Fig. 6:**
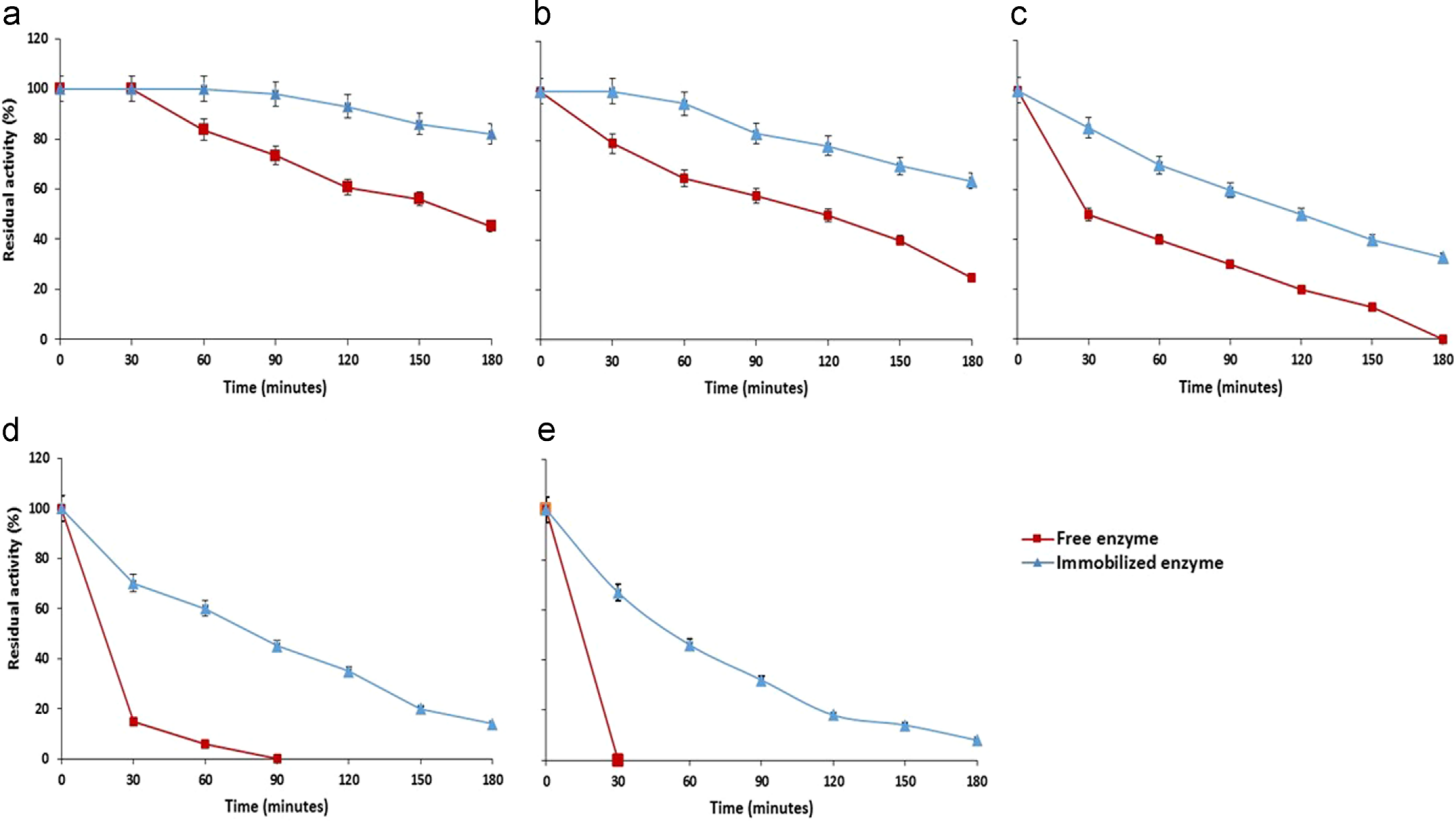
Thermal stability of free and calcium alginate immobilized maltase. (a) 40 °C, (b) 45 °C, (c) 50 °C, (d) 55 °C, and (e) 60 °C (means±S.E., *n*=6).

Thermal stability of free and immobilized maltase was also analyzed by keeping them at various temperatures ranging from 40 °C to 60 °C for different time intervals. Immobilized maltase showed broad stability range at different temperatures as compared to free enzyme (Fig. 6). Immobilized maltase retained 75% and 55% activity at 40 °C and 45 °C, respectively after 3.0 h whereas, free enzyme showed only 45% and 25% residual activity at the same temperatures. Free maltase lost its complete activity at 55 °C after 90.0 min. However, immobilized maltase retained its activity even after 180.0 min under same conditions. It was also observed that immobilized enzyme also retained 67% activity at 60 °C even after 30.0 min. Enzyme rigidity can be enhanced by immobilizing it in various supports where interaction between enzyme and matrix plays significant role to prevent the major conformational changes induced by the heat [28]. It was reported that various factors including number of bonds formed between enzyme and support, the nature of the bonds, the confinement degree of an enzyme within support and the environmental conditions of an enzyme may affects the stability of immobilized enzyme [29].

**Fig. 7:**
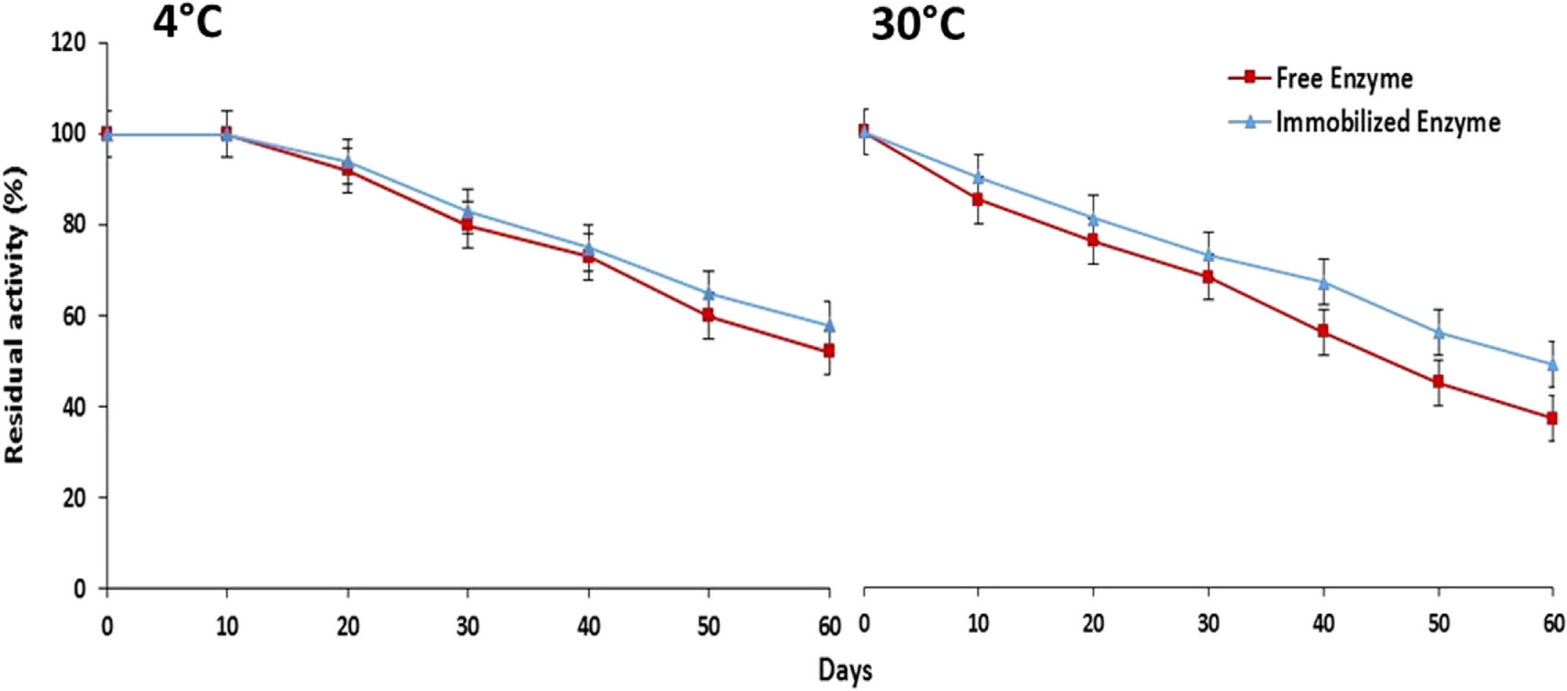
Storage stability of free and calcium alginate immobilized maltase (means±S.E., *n*=6).

Free and immobilized maltase were stored up to 60 days at different temperatures (4 °C and 30 °C) and residual activity was calculated. It was found that immobilized maltase showed broad storage stability range at 4 °C and 30 °C as compared to free enzyme (Fig. 7). Free maltase retained 52% and 37% residual activity at 4 °C and 30 °C, respectively. However, immobilized maltase showed 58% and 49% residual activity at same aforementioned storage conditions. Several research groups have reported that enzyme immobilization within hydrogel carriers like alginate, poly (hydroxyethylmethyacrylate-coglycidylmethacrylate), gelatin and polyacrylamide exhibited high stability due to the protective microenvironment provide by the gel matrix [3], [30], [31]. The current finding suggested that calcium alginate matrix generates positive impact by forming different interactions with an enzyme which also provides conformational stability to immobilized maltase.

**Fig. 8:**
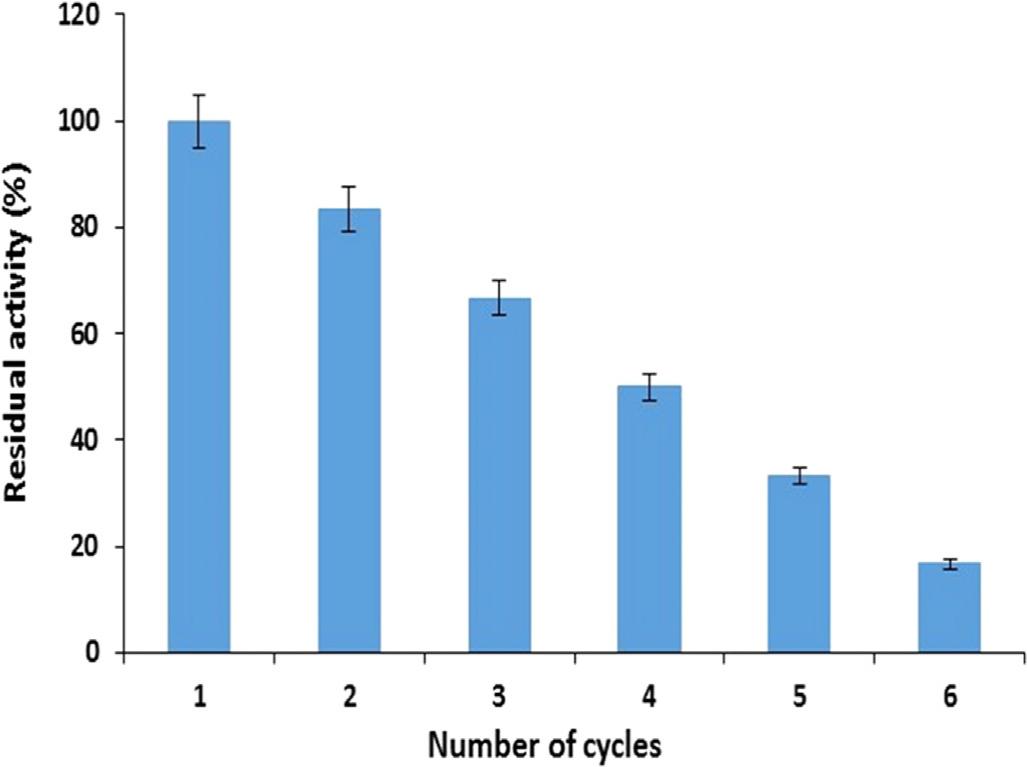
Recycling efficiency of calcium alginate immobilized maltase (means±S.E., *n*=6).

Immobilized maltase retained more than 80% and 67% recycling efficiency after 2nd and 3rd cycle. It was also observed that the immobilized maltase showed 17% residual activity even after 6th cycles (Fig. 8). The residual activity of immobilized maltase was decreased progressively as the number of cycles increased and complete loss of activity was found after six reaction cycles which might be due to the leakage of enzyme from support as a result of excessive washing of beads after each cycle. Similar results have been reported when enzyme was immobilized using calcium alginate as a support [15].

**Fig. 9:**
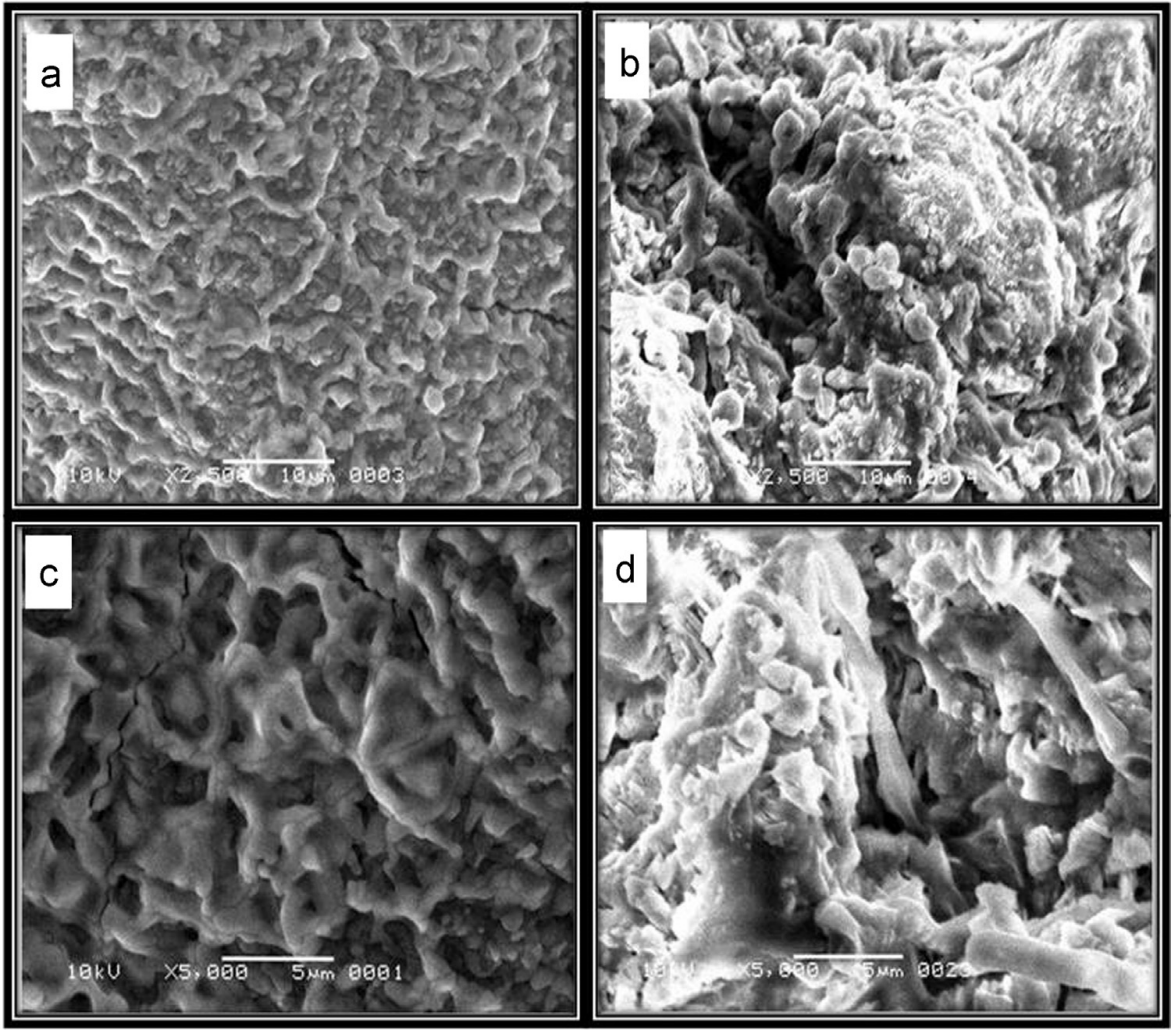
Scanning electron micrographs of calcium alginate beads before and after immobilization of maltase. (A and C) SEM micrographs of calcium alginate beads at 2500× and 500× of magnification before immobilization respectively. **(**B and D) SEM micrographs calcium alginate beads after immobilization of maltase 2500× and 5000× of magnification respectively.

The surface topology of calcium alginate beads before and after immobilization of maltase was examined by using scanning electron microscope (SEM). Compact pores was observed on the surface of beads having no enzyme (Fig. 9A and C) whereas, for maltase immobilized beads small particles with irregular shape were observed on the surface (Fig. 9B and D). Morphological changes on the surface of immobilized laccase were also observed where TiO_2_–montmorillonite complex was used as matrix [32].

## 4 Conclusions

This study justifies the use of calcium alginate as an appropriate support for the immobilization of maltase through entrapment technique. Immobilized enzyme showed remarkable thermal stability and recycling efficiency. All parameters of maltase were improved after immobilization which showed that enzyme stability and biocompatibility within calcium alginate matrix. Such improved catalytic efficiency of immobilized maltase is significant for various commercial applications.

## Conflict of interest

Authors declared no conflict of interest.

## References

[1] Mohamed R.A., Salleh A.B., Rahman R.N.Z.R.A., Basri M., Leow T.C. (2012). Afr. J. Microbiol. Res..

[2] Sarrouh B., Santos T.M., Miyoshi A., Dias R., Azevedo V. (2012). J. Bioprocess. Biotech.

[3] Dey G., Singh B., Banerjee R. (2003). Braz. Arch. Biol. Technol..

[4] Doaa A.R.M., Helmy W.A. (2009). J. Appl. Sci. Res..

[5] Homaei A.A., Sariri R., Vianello F., Stevanatom R. (2013). J. Chem. Biol..

[6] Jadhav S.B., Singhal R.S. (2014). Carbohydr. Polym..

[7] Talekar S., Ghodake V., Kate A., Samant N., Kumar C., Gadagkar S. (2010). Aust. J. Basic Appl. Sci..

[8] Bautista F.M., Bravo M.C., Campelo J.M., Garcia A., Luna D., Marinas J.M., Romero A.A. (1998). J. Chem. Technol. Biotechnol..

[9] Litjens M.J.J., Le K.Q., Straathof A.J.J., Jongejan J.A., Heijnen J.J. (2001). Biocatal. Biotransform..

[10] Kafshgari M.H., Khorram M., Mansouri M., Samimi A., Osfouri S. (2012). Iran. Polym. J..

[11] Kumari S., Panesar P.S., Bera M.B., Chopra H.K. (2014). Iran. Polym. J..

[12] Le-Tien C., Millette M., Lacriox M., Mateescu M.C. (2004). Biotechnol. Appl. Biochem..

[13] Matto M., Hussain Q. (2009). J. Mol. Catal. B: Enzym..

[14] Satar R., Matto M., Husain Q. (2008). J. Sci. Ind. Res..

[15] Rehman H.U., Aman A., Silipo A., Qader S.A.U., Molinaro A., Ansari A. (2013). Food Chem..

[16] Riaz A., Qader S.A.U., Anwar A., Iqbal S. (2009). Aust. J. Basic Appl. Sci..

[17] Nawaz M.A., Bibi Z., Aman A., Zohra R.R., Qader S.A.U. (2014). Pak. J. Pharm. Sci..

[18] Trinder P. (1969). J. Clin. Pathol..

[19] Trinder P. (1969). Ann. Clin. Biochem..

[20] Longo M.A., Novella. I.S., Garcia L.A., Daz M. (1992). Enzyme Microb. Tech..

[21] Ahmed S.A., Al Domany Ramadan A., El-Shayeb Nefisa M.A., Radwan H.H., Saleh S.A. (2008). S.A. Res. J. Agric. Biol. Sci..

[22] Waldman A.S., Schechinger L., Govindarajoo G., Nowick J.S., Pignolet L.H. (1998). J. Chem. Educ..

[23] Ertan F., Yagar H. (2007). Prep. Biochem. Biotechnol..

[24] Qader S.A.U., Aman A., Syed N., Bano S., Azhar A. (2007). Ital. J. Biochem..

[25] Bibi Z., Shahid F., Qader S.A.U., Aman A. (2015). Int. J. Biol. Macromol..

[26] Karim A., Qader S.A.U., Nawaz A., Aman A. (2014). Chem. Eng. Trans..

[27] Nawaz M.A., Karim A., Aman A., Marchetti R., Qader S.A.U., Molinaro A. (2014). Bioprocess Biosyst. Eng..

[28] Klibanov A.M. (1982). Adv. Appl. Microbiol..

[29] Chia M.C., Lyub R.C., Linb L.L., Huangc H.B. (2008). Biochem. Eng. J..

[30] Sahin F., Demirel G., Tumturk H. (2005). Int. J. Biol. Macromol..

[31] Yahsi A., Sahin F., Demirel G., Tumturk H. (2005). Int. J. Biol. Macromol..

[32] Wang Q., Peng L., Li G., Zhang P., Li D., Huang F., Wei Q. (2013). Int. J. Mol. Sci..

